# Outpatient Management of Gastroduodenal Artery Aneurysm

**DOI:** 10.7759/cureus.19091

**Published:** 2021-10-28

**Authors:** Ashley McGuire, Brianna Capron

**Affiliations:** 1 Family Medicine, Wellmont Health Systems, Norton, USA; 2 Family Medicine, University of Pikeville-Kentucky College of Osteopathic Medicine, Pikeville, USA

**Keywords:** gastrointestinal bleeding, abdominal pain, embolization, gastroduodenal artery, abdominal visceral aneurysm

## Abstract

Gastroduodenal artery (GDA) aneurysms are exceptionally rare. Although rare, providers should have a sufficient understanding of the causes, symptoms, and treatment options due to there being a high risk of mortality if these aneurysms rupture. It is important to understand the need for close management and follow-up in patients with this diagnosis, especially in the outpatient setting. Various treatment options are available and should be immediately discussed with every patient diagnosed with this aneurysm. We report a patient with a previously diagnosed gastroduodenal aneurysm who presented to the clinic with new-onset symptoms.

## Introduction

Common causes of gastroduodenal artery (GDA) aneurysms include pancreatitis, autoimmune disorders, blunt abdominal trauma, and infections [1]. Although a GDA aneurysm is a rare manifestation of these diseases, they can prove to be fatal. They account for 1.5% of all visceral artery aneurysms with an incidence rate of 0.01-0.2% and carry a 25% risk of rupture, leading to a 70% risk of death [2]. In the outpatient setting, it is important to recognize the most common symptoms associated with this condition; gastrointestinal bleeding, hematemesis, and abdominal pain [1]. Only 7.5% of GDA aneurysms are symptomatic [3] and may be found incidentally on imaging for other reasons. Regardless, all GDA aneurysms should be treated once diagnosed.

## Case presentation

A 53-year-old male presented to our primary care clinic in January 2021 for a three-month follow-up of chronic disease management with an acute complaint. Since awakening that morning, he experienced abdominal pain described as waxing and waning, sharp in intensity, lasting one to two minutes at a time, localized to his right upper quadrant. The patient denied nausea, vomiting, or hematemesis. Significant past medical/surgical history included atrial fibrillation, diabetes mellitus type 2, GI bleed secondary to marginal ulcer, obesity s/p gastric bypass, cholecystectomy, and hernia repair. Physical exam revealed stable vital signs and tenderness to palpation of the right upper quadrant. Upon further review of the relevant history, the patient revealed he was diagnosed with a GDA aneurysm in 2011. His GDA aneurysm was an incidental finding on a computed tomography (CT) scan he had done for unrelated reasons. There have been reports of GDA aneurysms occurring after gastric bypass surgery, however, this patient’s GDA aneurysm was diagnosed prior to his gastric bypass and prior to his hernia repair. Catheter ablation had been attempted previously at an outside facility but was unsuccessful and the patient had remained asymptomatic until this time.

CT angiogram of the abdomen, with and without contrast, performed in February 2019 showed the gastroduodenal artery to be “almost completely thrombosed with a minimal flow noted within the superior portion”. The size of the aneurysm was slightly increased in size to 2.3 cm × 3 cm compared to 2.2 cm × 2.7 cm in the CT angiogram from 2011.

Due to the patient's history and current symptoms, a Stat CT angiography of the abdomen with and without contrast was ordered to rule out changes that could indicate impending rupture or other possible complications. Pancreatitis is one of the most common risk factors associated with GDA aneurysms [1]. Amylase and lipase were ordered and found to be within normal limits. In addition to normal lab work, the pancreas did not show any acute or chronic findings on multiple CT scans, suggesting that pancreatitis was most likely not the cause of his aneurysm.

The Stat CT angiography revealed no evidence of rupture and no new aneurysms. The size was stable (around 3 cm) compared to the previous CT angiography from March 2019. It is described as partially thrombosed with calcified walls. Of interesting note, the patient was also found to have a 1.4 cm celiac artery aneurysm. 

**Figure 1:**
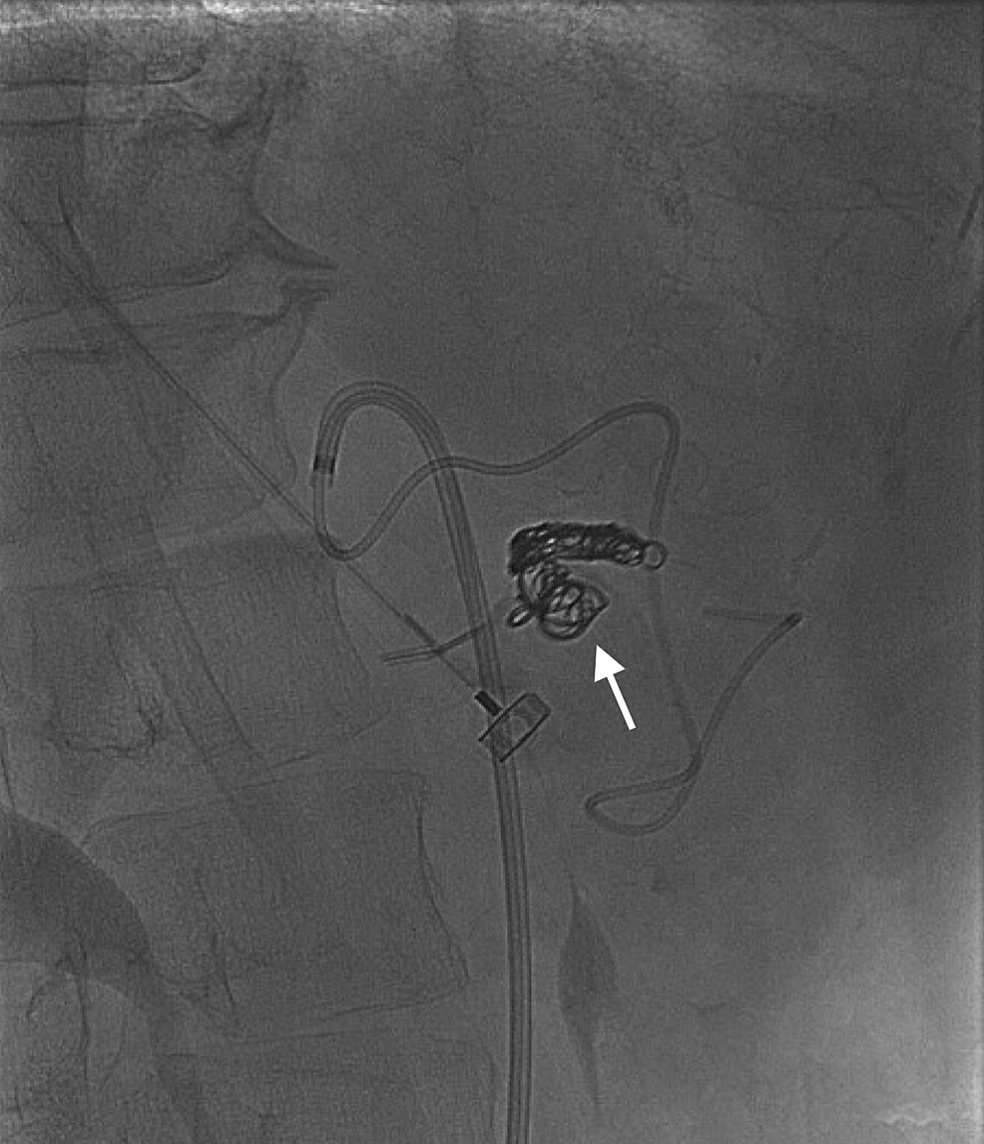
Successful coil embolization of gastroduodenal artery aneurysm. Arrow shows the patient’s coil embolization.

Referral was placed for vascular surgery for further evaluation, management, and treatment. The vascular surgeon determined that the GDA aneurysm required endovascular embolization. Coil embolization was performed and showed successful coiling of the aneurysm with no flow (Figure 1).

## Discussion

Gastroduodenal artery aneurysms are reported as extremely rare, and there is no standardized guideline on management of them due to a lack of studies. Patients may present with gastrointestinal bleeding, abdominal pain, or a pulsatile mass if there is a rupture. The most common risk factor associated with an increased risk of developing a GDA aneurysm is pancreatitis. The leakage of proteolytic enzymes that occur in pancreatitis results in erosion of the surrounding arteries causing aneurysms to form [2]. Other associated risk factors include blunt abdominal trauma, operative trauma during abdominal procedures, and autoimmune diseases causing inflammation of vascular walls leading to eventual aneurysm formation. Other diseases such as Marfan syndrome, Ehlers-Danlos syndrome, and fibromuscular dysplasia have been reported in a few cases as causes of pseudoaneurysms. Our patient did not have symptoms or history consistent with these diseases.

The gold standard for investigating gastrointestinal bleeding is esophagogastroduodenoscopy, but these may not always pick up a GDA aneurysm, as seen in some reported cases. If a GDA aneurysm is suspected, a visceral angiography is often used due to its high sensitivity compared to an abdominal CT (100% vs 76%) and can be therapeutic in addition to diagnostic [2]. Ultrasound can also be utilized with a sensitivity of 50% [2].

GDA aneurysms present with little to no symptoms and are often discovered incidentally. Once discovered, all GDA aneurysms, regardless of their size, must be treated due to their high potential to rupture. This patient was diagnosed 10-years prior and had remained asymptomatic with only a slight increase in the size of the GDA aneurysm. Current treatment options include surgical intervention (revascularization, vessel ligature, aneurysmal sac exclusion), or endovascular (coil embolization, stent placement) [1]. Transcatheter embolization has recently become an increasingly popular choice of treatment as opposed to surgical resection. Due to the difficulty associated with exposing the gastroduodenal artery, endovascular intervention is strongly preferred and may have lower postoperative complications [4,5]. Risks associated with endovascular repair include distal thromboembolic events, coil migration, intra-procedural aneurysm rupture, and non-target vessel embolization [6]. Close follow-up including postprocedure imaging to ensure the coiling was successful and to ensure there is no flow within the aneurysm should be performed.

In our case, it is unclear why our patient did not have surgical or endovascular intervention done at the initial time of diagnosis. Catheter ablation was attempted years after initial diagnosis but was unsuccessful and the patient was to be “followed closely”. Although his aneurysm remained stable for many years, the risk of rupture still existed. It is crucial to educate patients on the possible complications of their diagnoses. Patients should understand how important it is to continue to follow-up with their providers if the choice to observe rather than treat the aneurysm is made. GDA aneurysms may remain stable and asymptomatic but can ultimately rupture, turning fatal in an instant. Thankfully our patient’s new-onset abdominal pain was not due to rupture or complications and lead to successful treatment of the patient’s GDA aneurysm.

## Conclusions

Gastroduodenal artery aneurysms are rare and carry a significant risk of a life-threatening rupture. Although this condition is more commonly seen in patients with a history of chronic pancreatitis, it can occur in a multitude of settings. As in our case, it may only be encountered as an incidental finding. Regardless of the etiology, once identified, a GDA aneurysm warrants prompt treatment with endovascular repair as the preferred option to avoid catastrophe. This case helps provide guidance to providers who are faced with the task of managing GDA aneurysms in the outpatient setting. Appropriate management includes discussing the risk and benefits of treatment with the patient and emphasizing the risks of rupture and death if untreated.
